# Global Profiling of Protein Lysine Lactylation in Mouse Cardiac Hypertrophy: A Lactylome Analysis

**DOI:** 10.3390/jcdd13070297

**Published:** 2026-06-29

**Authors:** Wengen Zhu, Siyu Guo, Yunyao Yang, Yugang Dong, Chen Liu, Cong Chen

**Affiliations:** 1 Department of Cardiology, The University of Hong Kong-Shenzhen Hospital, Shenzhen 518053, China; zhuwg6@mail.sysu.edu.cn; 2 Department of Cardiology, The First Affiliated Hospital of Sun Yat-Sen University, Guangzhou 510080, China; guosy9@mail2.sysu.edu.cn (S.G.); yyyniceday@163.com (Y.Y.); dongxg@mail.sysu.edu.cn (Y.D.); 3 NHC Key Laboratory of Assisted Circulation and Vascular Diseases, Sun Yat-Sen University, Guangzhou 510080, China; 4 National-Guangdong Joint Engineering Laboratory for Diagnosis and Treatment of Vascular Diseases, Guangzhou 510080, China

**Keywords:** heart failure, cardiac hypertrophy, lysine lactylation, mitochondria, sarcomere

## Abstract

Background: Cardiac hypertrophy, a major feature of heart failure, is closely linked to metabolic remodeling and energy deficiency. Lysine lactylation (Kla), a recently discovered post-translational modification (PTM), has been implicated in various cellular processes. However, its specific role in cardiac hypertrophy remains poorly understood. Methods: We conducted quantitative proteomics and Kla PTM analysis on left ventricular tissues from both sham-operated and aortic banding-induced hypertrophic mouse hearts. Protein samples were extracted, enriched for lactylation, and subjected to mass spectrometry. Bioinformatic analyses were performed to uncover pathways and protein–protein interactions (PPI) related to Kla-modified proteins. Results: Our lactylome analysis identified 159 Kla-modified sites across 80 proteins, with 72 proteins exhibiting elevated Kla levels, particularly in mitochondrial and sarcomeric proteins. Pathway enrichment analysis highlighted significant involvement of fatty acid metabolism, the tricarboxylic acid (TCA) cycle, and cardiomyopathy-related pathways, underscoring the role of Kla in energy metabolism and cardiac remodeling. PPI analysis further revealed the central role of metabolic and structural proteins in the hypertrophic response. Conclusions: Our study provides the comprehensive analysis of Kla in cardiac hypertrophy, revealing its significant role in modulating proteins involved in mitochondrial energy metabolism and sarcomeric structure. Our findings provide a comprehensive overview of the lactylation landscape in cardiac hypertrophy and reveal extensive lactylation changes in proteins associated with mitochondrial metabolism and sarcomeric organization. These observations suggest a potential link between Kla and cardiac hypertrophy, which warrants further functional investigation.

## 1. Introduction

Heart failure is a serious and often terminal stage of many cardiovascular diseases [1,2], contributing to high morbidity and mortality rates despite advancements in medical management. Current therapies often fail to significantly improve patient outcomes, underscoring the need for further understanding of heart failure pathogenesis and the development of novel therapeutic approaches. A key driver of heart failure is cardiac hypertrophy [3,4], characterized by the enlargement of heart muscle cells and an increase in overall heart mass. This condition typically arises from chronic pressure overload, leading to significant metabolic alterations within the heart. Both fatty acid and glucose oxidation pathways are disrupted, resulting in an energy imbalance that accelerates the progression toward heart failure [5,6,7].

Recent research has indicated the role of mitochondrial energy production and lactate metabolism in cardiac hypertrophy [8,9,10]. Lactate, previously considered a mere byproduct of glycolysis [11,12], is now recognized as a crucial signaling molecule involved in regulating cardiac hypertrophy. However, the exact mechanisms through which lactate affects the heart are not fully understood. One emerging hypothesis is the involvement of lysine lactylation (Kla), a novel post-translational modification (PTM) that regulates gene expression and protein function in various cellular processes [13,14].

Kla has been implicated in diverse pathological conditions [15,16,17,18,19,20,21,22], including cancer and inflammatory diseases, and there is growing evidence of its role in cardiovascular diseases [15,23,24,25,26,27]. Specifically, Kla influences processes such as inflammation, fibrosis, and cardiac contractility. However, its precise function in cardiac hypertrophy and the transition to heart failure remains largely unexplored.

Given that lactate accumulation is a common feature in hypertrophic cardiomyocytes due to enhanced glycolytic flux, it is critical to determine whether Kla plays a role in the development of cardiac hypertrophy. This study aims to address this gap by performing quantitative proteomic and Kla PTM analyses in a mouse model of cardiac hypertrophy induced by aortic banding (AB). 

## 2. Materials and Methods

### 2.1. Animals

The C57BL/6J mice, aged 8 to 10 weeks and weighing 24 to 26 g, were bred at the Animal Experiment Center of Sun Yat-sen University. All procedures adhered to the guidelines outlined in the Guide for the Care and Use of Laboratory Animals published by the US National Institutes of Health and were approved by the Animal Care and Use Committees of Sun Yat-sen University. Cardiac hypertrophy was induced through pressure overload by performing descending aortic banding (AB), while sham-operated wild-type (WT) mice served as controls. The surgical procedures were conducted as previously described [28,29,30].

To minimize biological variability associated with the banding model, age- and body weight-matched mice were used. Male mice were included in this study. The AB procedure was maintained for 4 weeks before tissue collection. Before sacrifice, mice were evaluated for evidence of cardiac hypertrophy based on heart weight/body weight ratio, heart weight/tibia length ratio, and echocardiographic parameters. Cardiac function was assessed by echocardiography measurements, including left ventricular ejection fraction, fractional shortening and other parameters. Only mice showing successful pressure-overload-induced cardiac hypertrophy were included in the proteomic and lactylome analyses.

### 2.2. Neonatal Rat Ventricular Myocyte Culture and Treatment

Neonatal rat ventricular myocytes were isolated from 1–2 day-old Sprague-Dawley rats as previously described [31]. Cells were cultured in DMEM supplemented with 10% fetal bovine serum and 1% penicillin-streptomycin. After 48 h, cells were treated with 50 μM phenylephrine for 24 h to induce hypertrophic responses. Protein extraction followed the same protocol as for mouse tissues.

### 2.3. Protein Extraction and Trypsin Digestion

Proteins were extracted from left ventricular tissues from both AB and WT mice. The sample was finely ground into cell powder using liquid nitrogen and transferred to a 5-mL centrifuge tube. Four volumes of lysis buffer (8 M urea and 1% protease inhibitor cocktail) were then added to the cell powder, followed by sonication on ice using a high-intensity ultrasonic processor (Scientz, Ningbo, China) in three intervals. The mixture was centrifuged at 12,000 *g* for 10 min at 4 °C to pellet the debris. The supernatant was carefully collected, and the protein concentration was determined using a bicinchoninic acid (BCA) kit, according to the manufacturer’s instructions.

For protein digestion, the solution was first reduced with 5 mM dithiothreitol at 56 °C for 30 min, followed by alkylation with 11 mM iodoacetamide at room temperature for 15 min in the dark. The protein sample was then diluted with 100 mM TEAB (triethylammonium bicarbonate) to lower the urea concentration to below 2 M. Trypsin was added at a 1:50 enzyme-to-protein mass ratio for an overnight digestion, followed by a second digestion at a 1:100 ratio for 4 h. The resulting peptides were desalted using a C18 solid-phase extraction (SPE) column.

### 2.4. Western Blot

Proteins (20 µg) were separated using SDS-PAGE and transferred to PVDF membranes. Membranes were blocked with 5% skim milk in TBS-T (1× TBS, 0.1% Tween-20) for 1 h at room temperature and incubated overnight at 4 °C with pan-anti-lactyl (pan-Kla) Lysine Rabbit pAb (No. PTM-1401, PTMBIO, 1:1000 dilution). After washing three times with TBS-T, membranes were incubated with goat anti-rabbit IgG (H + L) secondary antibody (1:10,000 dilution) at room temperature for 1 h. Protein bands were visualized using an enhanced chemiluminescence kit (No. 35055, Thermo Scientific, Waltham, MA, USA).

### 2.5. Pan-Antibody-Based PTM Enrichment

Fractionated peptides were dissolved in NETN buffer (100 mM NaCl, 1 mM EDTA, 50 mM Tris, 0.5% NP40, pH 8.0) and incubated with pan-Kla antibody-conjugated agarose beads overnight at 4 °C with gentle shaking. Beads were washed four times with NETN buffer and twice with ddH_2_O, followed by elution with 0.1% trifluoroacetic acid. Eluted peptides were combined, vacuum-dried, and desalted with C18 ZipTips (Millipore, Burlington, MA, USA).

### 2.6. Proteome and Lactylome Analysis by LC-MS/MS

Four-dimensional label-free lactylation quantitative proteomic analysis was performed by Jingjie PTM BioLab Co., Ltd. (Hangzhou, China). Pan-Kla antibody-conjugated agarose beads were used to enrich lactylated proteins. Global proteomics and Kla-enriched lactylome analyses were performed from the same biological samples but represented two distinct analytical workflows. For global proteomic profiling, non-enriched peptide fractions were directly subjected to LC-MS/MS (Liquid Chromatography-Tandem Mass Spectrometry) to assess overall protein abundance. For lactylome profiling, peptide fractions were first enriched using pan-Kla antibody-conjugated agarose beads, and the enriched Kla-containing peptides were then analyzed by LC-MS/MS to identify and quantify lactylated sites.

Peptides were dissolved in 0.1% formic acid in water (solvent A) and loaded onto a homemade reversed-phase analytical column. The gradient of solvent B (0.1% formic acid in acetonitrile) was 6–24% over 70 min, 24–35% in 14 min, increased to 80% in 3 min, and held at 80% for 3 min at a flow rate of 450 nL/min on a nanoElute UHPLC system (Bruker Daltonics, Billerica, MA, USA). Peptides were analyzed using a timsTOF Pro mass spectrometer (Bruker Daltonics, Billerica, MA, USA) operated in PASEF mode with 10 PASEF-MS/MS scans per cycle.

For quality control, reproducibility was evaluated separately for the global proteomic dataset and the Kla-enriched lactylome dataset. Pearson correlation analysis, principal component analysis, and relative standard deviation analysis were performed for each dataset independently. The corresponding quality-control results have been clearly labeled as global proteomics or Kla-enriched lactylome data in the revised Supplementary Figure S2.

### 2.7. Database Search

Raw data were processed with MaxQuant (v.1.6.15.0) against the mouse SwissProt database (concatenated with a reverse decoy database). Trypsin specificity with up to two missed cleavages was set. Carbamidomethylation of cysteine was set as a fixed modification, and oxidation of methionine and N-terminal acetylation were set as variable modifications. False discovery rate (FDR) was adjusted to <1%.

### 2.8. Bioinformatics Analysis

The proteome was annotated with Gene Ontology (GO) terms from the UniProt-GOA database (http://www.ebi.ac.uk/GOA/ accessed on 13 February 2023), categorizing proteins into three main classifications: biological processes, cellular components, and molecular functions. For proteins not covered by UniProt-GOA (version 2.2), InterProScan (5.60-92.0) was used to perform sequence-based annotations. Subcellular localization predictions were made using Wolfpsort (0.2) for eukaryotic proteins, and motif analysis was carried out with the MoMo tool (5.5.1). Protein domains were annotated via InterProScan, leveraging the InterPro database for functional insights. The Kyoto Encyclopedia of Genes and Genomes (KEGG) pathway annotation was performed using the KAAS tool (105.0), with subsequent mapping conducted through KEGG Mapper (105.0). Functional enrichment analysis for GO and KEGG terms was carried out using a two-tailed Fisher’s exact test, with terms considered significant at *p* < 0.05. Hierarchical clustering of differentially expressed proteins, based on enrichment results such as GO, Domain, Pathway, and Complex, was visualized as heat maps. Protein–protein interaction (PPI) networks were constructed using STRING (v11.0) with a confidence score threshold of ≥0.7 and visualized with the R package “networkD3”.

### 2.9. Data Analysis

The fold change (FC) was calculated by comparing the mean relative quantitative values of the modified Kla sites in the AB group to those in the WT group. Modified Kla sites were considered differentially regulated if the FC was greater than 1.5 (upregulated) or less than 1/1.5 (downregulated). Protein abundance between the AB and WT groups was assessed using quantitative data. Missing values were imputed using a minimum value approach for lactylome data. Normalization was performed based on total peptide intensity. For differential analysis, *p*-values were adjusted using the Benjamini–Hochberg false discovery rate (FDR) correction; sites with FDR < 0.05 and fold change > 1.5 were considered significant.

## 3. Results

### 3.1. Protein Collection and Mass Spectrometry Workflow in Cardiac Hypertrophy Models

**Figure 1:**
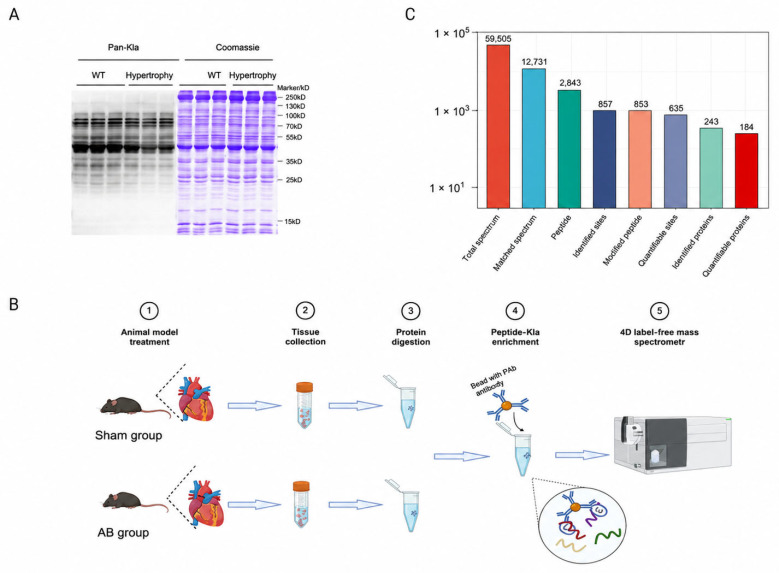
Protein Collection and Mass Spectrometry Workflow in Cardiac Hypertrophy Models. (**A**) Immunoblots and Coomassie Brilliant Blue staining demonstrating protein lactylation levels between cardiac hypertrophy and sham-operated WT mice (n = 3 hearts per genotype). (**B**) Schematic representation of the mass spectrometry workflow, from sample collection through protein extraction to mass spectrometry analysis. (**C**) Total spectra obtained from liquid chromatography-coupled 4D label-free mass spectrometry analysis. Abbreviations: Kla = lysine lactylation; WT = wild type; AB = aortic banding.

We collected total protein samples from in vivo and in vitro models of cardiac hypertrophy: left ventricular tissues from AB mice and neonatal rat ventricular myocytes treated with 50 μM phenylephrine (Supplementary Figure S1), respectively. To assess protein Kla levels, we used a pan-Kla antibody for immunoprecipitation and compared Kla levels between AB and sham-operated WT mice. Immunoblotting results (Figure 1A) showed differences in Kla levels between groups. For the proteomics and Kla PTM omics analyses, three mice per group were used as biological replicates. To ensure reproducibility, we conducted several quality control analyses, including Pearson’s correlation, principal component analysis (PCA), and relative standard deviation (RSD) (Supplementary Figure S2), all of which confirmed the reliability of our data.

The experimental workflow (Figure 1B) outlines the process from sample collection to LC-MS/MS. In total, 857 lactylated sites in 243 proteins were identified, of which 635 sites in 184 proteins were quantified (Figure 1C). We conducted control analyses to ensure data quality, including evaluations of peptide length, peptide count per protein, protein coverage, and molecular weight distribution (Supplementary Figure S3). Most peptides ranged from 7 to 20 amino acids, protein coverage was below 30%, and the identified proteins showed a wide range of molecular weights, all indicating high data quality.

### 3.2. Functional Classification Analysis of Kla Proteins in Cardiac Hypertrophy

**Figure 2:**
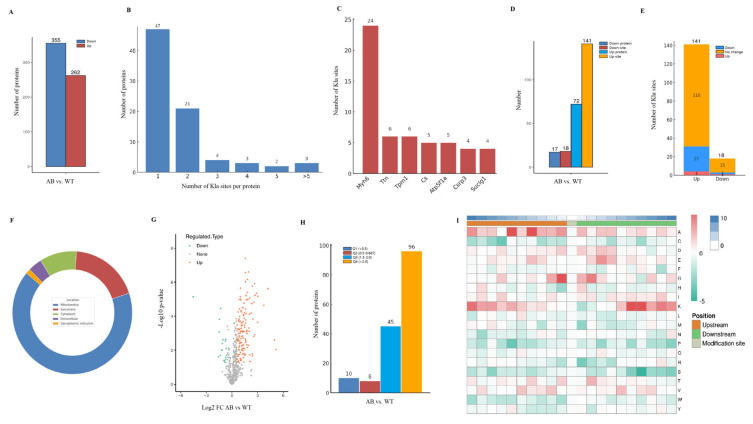
Differential Kla Modifications and Subcellular Localization in Cardiac Hypertrophy. (**A**) Differential expression of proteins in AB vs. sham-operated WT mice. (**B**) Distribution of proteins based on the number of Kla modification sites. (**C**) Number of Kla modification sites identified across the top 7 proteins. (**D**) Number of proteins with significant Kla level changes at lysine modification sites. (**E**) Relationship between Kla level alterations and protein expression levels. (**F**) Subcellular localization of differentially Kla-modified proteins. (**G**) Volcano plot showing differentially modified Kla sites. (**H**) Clusters of differentially Kla-modified proteins based on expression levels. (**I**) Motif analysis of Kla sites using the Motif-X-based MoMo tool. Abbreviations: Kla = lysine lactylation; WT = wild type; AB = aortic banding.

Our analysis identified 355 proteins that were downregulated and 262 proteins that were upregulated in AB mice compared to sham-operated WT controls (Figure 2A). Using antibody-based enrichment and LC-MS/MS, we found 159 significantly modified Kla sites across 80 proteins, with the majority (58.75%) of proteins showing only a single Kla modification site (Figure 2B and Table 1). Myosin heavy chain 6 (Myh6) had the highest number of Kla modification sites (n = 24), followed by tropomyosin alpha-1 (Tpm1) and titin (Ttn) with six sites each (Figure 2C).

A total of 72 proteins showed significantly increased Kla levels at 141 lysine sites (Figure 2D). Among these, 17 proteins exhibited a decrease in overall protein levels, but Kla levels still increased at 27 sites within these proteins, while one protein showed an overall increase in abundance, with Kla levels rising at four sites (Figure 2E). Conversely, 17 proteins experienced a significant decrease in Kla levels at 18 lysine sites. Among these, one protein showed an overall increase in protein levels, with Kla levels deceasing at one site, while another protein exhibited a decrease in both overall protein levels and Kla levels at two sites.

Next, we analyzed the subcellular localization, suggesting that the majority of these 80 modified proteins were located in the mitochondria (66.3%), followed by the sarcomere (18.8%) and the cytoplasm (10.0%) (Figure 2F). The volcano plot (Figure 2G) highlights the differentially modified Kla sites, with most sites (n = 96) falling within Q4 (i.e., fold change ≥ 2.0), indicating significant upregulation of Kla sites in AB mice (Figure 2H).

Subsequently, we conducted GO analysis to categorize the differentially Kla-modified proteins based on biological processes, cellular components, and molecular functions. The GO analysis of differentially Kla-modified proteins reveals that the majority are involved in cellular processes (74 proteins) and metabolic processes (58 proteins) under biological processes. In terms of cellular components, these proteins are primarily associated with intracellular and cellular locations (79 proteins each), and in the molecular function category, binding (55 proteins) and catalytic activity (47 proteins) are the most enriched functions (Supplementary Figure S4). Further functional analysis of differentially Kla-modified proteins based on Clusters of Orthologous Groups (COG/KOG) categories (Supplementary Figure S5) showed a predominant enrichment in metabolism-related categories, particularly in energy production and conversion (36 proteins), followed by cellular processes such as cytoskeleton structure (12 proteins). Energy production and cytoskeletal structure were the main functions of these proteins. The GO and COG/KOG analysis for upregulated and downregulated Kla modified proteins are shown in Supplementary Figures S6–S9.

### 3.3. Functional Enrichment Analysis of Kla Proteins in Cardiac Hypertrophy

**Figure 3:**
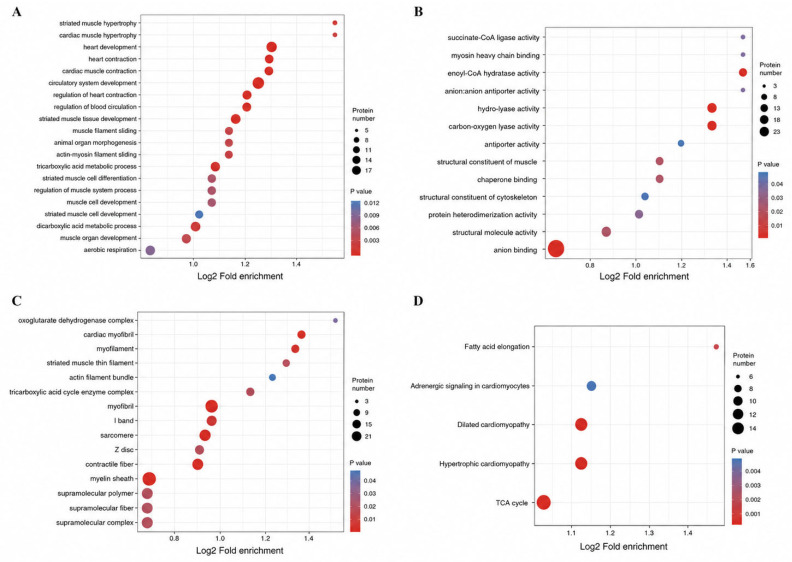
GO and KEGG Functional Enrichment of Kla-modified Proteins in Cardiac Hypertrophy. (**A**) Enrichment of Kla-modified proteins in biological processes related to cardiac muscle function and development. (**B**) Enrichment of Kla-modified proteins in molecular functions related to muscle structure and energy metabolism. (**C**) Kla-modified proteins enriched in cellular components, including myofilaments and TCA cycle enzyme complexes. (**D**) KEGG pathway enrichment analysis showing the involvement of Kla-modified proteins in metabolic and cardiac pathways. Abbreviations: Kla = lysine lactylation; TCA = tricarboxylic acid; GO = Gene Ontology; KEGG = Kyoto Encyclopedia of Genes and Genomes.

To explore the functions of Kla-modified proteins, we performed GO and KEGG pathway enrichment analysis. In terms of biological processes (Figure 3A), differentially Kla-modified proteins were predominantly involved in muscle and cardiac function, such as striated muscle hypertrophy, heart development, and cardiac muscle contraction. At the molecular level, Kla modifications were enriched in processes related to muscle structure and energy metabolism, such as myosin heavy chain binding, enoyl-CoA hydratase activity, and succinate-CoA ligase activity (Figure 3B), suggesting that Kla modifications may affect both structural integrity and metabolic efficiency in the heart. As shown in Figure 3C, Kla-modified proteins were enriched in cellular components crucial to muscle structure and energy metabolism, such as myofilaments, Z disc, and the tricarboxylic acid (TCA) cycle enzyme complex, suggesting that Kla plays a role in both the structural components of muscle cells and their energy production machinery.

The KEGG pathway enrichment analysis revealed significant enrichment in pathways such as dilated cardiomyopathy, hypertrophic cardiomyopathy, and the TCA cycle (Figure 3D). The GO and KEGG functional enrichment analysis for upregulated and downregulated Kla modified proteins are shown in Supplementary Figures S10 and S11, suggesting that upregulated Kla modified proteins are closely related to energy metabolism and sarcomeric structure in cardiac hypertrophy.

To comprehensively understand the effects of Kla modification in cardiac hypertrophy, the Kla-modified proteins were divided into four clusters based on their expression levels (Q1: <0.5, Q2: 0.5–1/1.5, Q3: 1.5–2.0, Q4: >2.0; Figure 2F). The KEGG pathway enrichment analysis indicated that the TCA cycle enriched in the most highly upregulated group (Q4), and cardiomyopathy pathways enriched in moderately upregulated proteins (Q3) (Supplementary Figure S12).

To further investigate the characteristics of Kla sites, we utilized the Motif-X-based MoMo analysis tool to examine the flanking sequences from −10 to +10 around the Kla site. The analysis revealed that alanine (A), aspartic acid (D), glycine (G), and histidine (H) were enriched at the +1 position, while alanine (A), aspartic acid (D), glycine (G), and threonine (T) were commonly found at the −1 position. Notably, glycine (G) at the −1 position exhibited the highest frequency (Figure 2I), suggesting its potential importance in influencing or stabilizing the modification at the Kla site due to its prominence in the surrounding sequence.

### 3.4. PPI Network Analysis of Kla Proteins in Cardiac Hypertrophy

**Figure 4:**
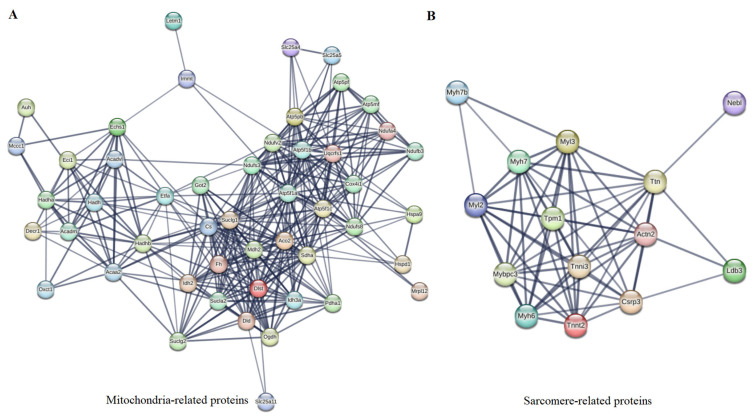
Protein–protein interaction Network Analysis of Kla Proteins in Cardiac Hypertrophy. (**A**) Protein–protein interaction network of mitochondria-related Kla proteins involved in cardiac hypertrophy. (**B**) Protein–protein interaction network of sarcomere-related Kla proteins involved in cardiac hypertrophy. Abbreviations: Kla = lysine lactylation.

Supplementary Figure S13 presents the PPI network of all Kla proteins implicated in cardiac hypertrophy. The overall network revealed two distinct clusters of proteins: one related to mitochondrial function and energy production, including proteins such as ATP synthase F1 subunit beta (Atp5b) and Cytochrome c oxidase subunit 4 isoform 1 (Cox4i1), and the other consisting of sarcomere-related proteins, such as Ttn, myosin heavy chain 7 (Myh7), and cardiac troponin T (Tnnt2), crucial for maintaining cardiac structure and contraction (Figure 4A,B). Together, these findings emphasize the critical roles of both mitochondrial dysfunction and structural abnormalities in cardiac hypertrophy.

## 4. Discussion

**Figure 5:**
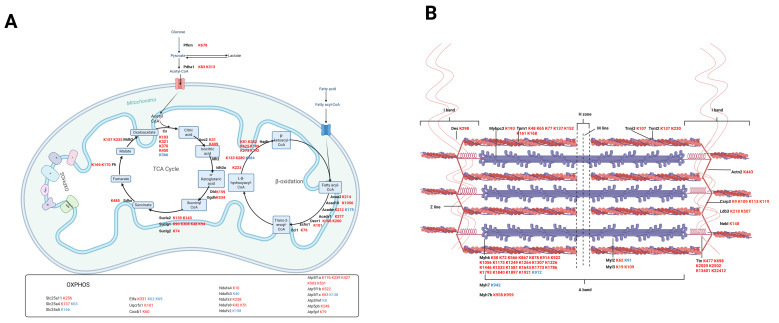
Kla proteins and sites in mitochondrial metabolism and sarcomeric structure during cardiac hypertrophy. (**A**) Kla-modified proteins in the TCA cycle and β-oxidation pathways. (**B**) Kla modifications in key sarcomeric proteins, including myosin and troponins, involved in cardiac contraction. Abbreviations: Kla = lysine lactylation; TCA = tricarboxylic acid.

In this study, we performed the comprehensive lactylome analysis of Kla in cardiac hypertrophy, uncovering significant modifications in proteins linked to mitochondrial energy metabolism and sarcomeric structure (Figure 5). These findings reveal extensive Kla modifications in proteins involved in metabolic and structural pathways, suggesting that Kla may be associated with cardiac remodeling processes.

### 4.1. Lactylation and Metabolic Reprogramming in Cardiac Hypertrophy

Cardiac hypertrophy is a pathological response to increased mechanical stress, such as pressure overload, and is characterized by a shift in metabolic pathways [4,6,32]. Normally, the heart relies heavily on fatty acid oxidation for ATP production, but during hypertrophy, there is a well-documented switch towards glycolysis, a phenomenon known as “metabolic reprogramming” [33,34]. Although glycolysis provides a rapid source of ATP, this shift is often insufficient to meet the heightened energy demands of hypertrophic cardiomyocytes, contributing to long-term energy deficits that accelerates the progression towards heart failure. Our data suggest that numerous mitochondrial metabolic enzymes undergo lactylation during cardiac hypertrophy, raising the possibility that Kla may participate in metabolic remodeling, particularly those involved in the TCA cycle and fatty acid β-oxidation pathways, thus influencing energy production in hypertrophic hearts.

The identification of lactylation sites on key metabolic enzymes such as isocitrate dehydrogenase (Idh2/3) [35,36] and hydroxyacyl-CoA dehydrogenase (Hadha/b) links to the potential mechanism where Kla modulates the activity of these enzymes, thereby influencing energy production. Specifically, lactylation of Idh2/3 identifies these enzymes as potential targets of Kla and raises the possibility that lactylation may influence TCA cycle activity. However, direct effects on enzyme activity and ATP production would be experimentally validated. Similarly, lactylation of Hadha/b suggests a regulatory role in lipid metabolism. This regulatory role of Kla on mitochondrial function opens new avenues for understanding how PTMs directly impact energy homeostasis, particularly in cardiac hypertrophy, where efficient energy production is critical for cell survival and function. Future studies should focus on exploring the functional consequences of Kla on these enzymes, as targeting Kla might improve mitochondrial efficiency and slow heart failure progression.

### 4.2. Lactylation and Structural Remodeling in Cardiac Hypertrophy

Beyond metabolism, Kla was notably enriched in sarcomeric proteins, such as Ttn, Myh7, and Tnnt2, which are critical for maintaining cardiac contractility and structure [37]. These proteins also serve as genetic markers in hypertrophic cardiomyopathy [37,38,39,40], a condition closely linked to cardiac hypertrophy. The lactylation of these structural proteins suggests a role for Kla in maintaining sarcomeric integrity under mechanical stress.

Previous studies have demonstrated that lactate and lactylation influence protein stability and gene expression, particularly under conditions of mechanical or metabolic stress [9,15,41]. Given that mutations in sarcomeric proteins are commonly associated with hypertrophic cardiomyopathy [37,42], it is plausible that lactylation at sarcomeric proteins represents a compensatory mechanism aimed at preserving sarcomeric integrity in hypertrophic hearts. We observed extensive Kla modifications on multiple sarcomeric proteins, including Myh6, Tpm1, Ttn, Tnni3 and Tnnt2. These findings suggest that sarcomeric proteins are major targets of lactylation during cardiac hypertrophy. However, whether these modifications influence protein stability, sarcomere organization, or contractile function requires further investigation. 

### 4.3. Lactylation as a Regulatory Mechanism in Cardiac Diseases

The identification of extensive Kla modifications expands the role of lactate from a metabolic byproduct to a critical signaling molecule in cardiovascular disease. Kla could act as a “metabolic switch” that regulates cellular stress responses, influencing a broad range of processes from energy metabolism to gene expression and structural adaptation [11,41,43]. In conditions such as myocardial infarction and heart failure [23,24,27], elevated lactate levels have been shown to correlate with worse outcomes, and interventions targeting lactate metabolism have begun to show promise in experimental models. However, it remains unclear whether Kla serves as an adaptive mechanism in early hypertrophy or contributes to disease progression when dysregulated. We have demonstrated that Kla modifications are significantly enriched on mitochondrial metabolic enzymes and sarcomeric structural proteins in hypertrophic hearts. In the context of pressure overload, the heart undergoes metabolic remodeling and struggles to preserve contractile function. Lactylation of TCA cycle enzymes and fatty acid oxidation proteins could potentially represent an adaptive response to optimize energy production. Similarly, lactylation of sarcomeric proteins might stabilize the cardiac structure during remodeling process. This speculation is based on the known functions of the modified proteins in supporting cardiac energetics and structure during the compensatory phase of hypertrophy [44]. However, we acknowledge that without functional validation, it remains unclear whether prolonged or dysregulated Kla contributes to maladaptive processes. Future studies employing site-specific mutations and functional experiments are essential to determine whether Kla is pro-adaptive, maladaptive, or context-dependent.

Additionally, further research should investigate how Kla interacts with other PTMs such as phosphorylation and acetylation, which are known to regulate both metabolism and structural proteins in the heart. Understanding these interactions will be key to deciphering the full regulatory scope of Kla in cardiac diseases and identifying therapeutic strategies to restore homeostasis in hypertrophic hearts. Although our findings have revealed novel lactylation targets and their potential association between cardiac hypertrophy, the definitive mechanism remains unclear and would be identified by functional validation.

### 4.4. Lactylation as a Regulatory Mechanism in Cardiac Diseases

Previous cardiac lactylome studies have mainly focused on myocardial infarction and neonatal cardiac regeneration, whereas the present study investigated pressure-overload-induced cardiac hypertrophy. Wu et al. [45] characterized protein lactylation in healthy and ischemic mouse hearts and reported extensive lactylation changes after myocardial infarction including altered lactylation of metabolic enzymes, cytoskeletal proteins, and RNA-binding proteins. In this study, myosin-6 and titin were among the proteins with the highest numbers of lactylation sites, and infarcted hearts showed 61 upregulated lactylation sites across 53 proteins and 30 downregulated sites across 27 proteins [45]. Zhang et al. analyzed postnatal mouse hearts at P1, P5, and P7 and identified 2297 Kla sites from 980 proteins, among which 1262 sites from 409 proteins were quantified. This study connected dynamic protein lactylation to metabolic reprogramming during the developmental process in which myocardial regenerative capacity is progressively lost [46].

Compared with previous studies, our pressure-overload model showed both similar and model-specific characteristics. Similarly to the myocardial infarction and neonatal regeneration datasets, our lactylome was enriched in proteins involved in mitochondrial metabolism, fatty acid oxidation, the TCA cycle, and sarcomeric organization. These overlapping categories suggest that metabolic and structural proteins may represent common cardiac lactylation targets across distinct processes of cardiac remodeling. However, the biological context is substantially different among these three models. Myocardial infarction is predominantly characterized by acute ischemic injury and tissue repair, neonatal cardiac remodeling is associated with developmental metabolic transition and loss of regenerative capacity, whereas AB-induced hypertrophy reflects chronic pressure overload and compensatory-to-maladaptive process. Thus, the potential biological function of Kla may not be identical in different models.

In our dataset, differentially lactylated proteins were particularly enriched in mitochondrial and sarcomeric compartments, with Myh6, Tpm1, Ttn, Tnni3, and Tnnt2 showing multiple Kla sites. These findings partially overlap with previous reports identifying myosin and titin as major lactylated cardiac proteins, but our study extends the current understanding of cardiac lactylation to pressure-overload-induced hypertrophy. Importantly, failing hearts undergo the transition to a fetal or neonatal metabolic program, including reduced reliance on fatty acid oxidation and increased glycolytic remodeling. The comparison with neonatal cardiac lactylome data also suggests that lactylation may be involved in metabolic remodeling across developmental and pathological contexts. However, the direct association among pressure overload, myocardial infarction, and neonatal regeneration should not be assumed. Future studies integrating raw datasets across models and site-specific functional validation would be performed to determine whether Kla sites are conserved or context-dependent in cardiac remodeling.

## 5. Limitation and Future Directions

The enzymes responsible for regulating Kla in the heart, such as lactyltransferases (writers), delactylases (erasers), and Kla-binding proteins (readers), remain largely unidentified. Recent studies have implicated enzymes such as alanyl-tRNA synthetase (AARS1) [47,48], histone Acetyltransferase 1 (HBO1) [49], lysine Acetyltransferase 8 (KAT8) [50], and Nijmegen Breakage Syndrome 1 (NBS1) [51] in lactylation, but it remains unclear whether these or other enzymes are involved in regulating lactylation in the heart. Identifying these enzymes will be crucial for understanding the dynamic regulation of Kla in response to metabolic and mechanical stress. Additionally, further exploration of the interplay between Kla and other metabolic processes, like the lactate shuttle and mitochondrial pyruvate transport, could reveal more about Kla’s role in heart disease. Future studies would include functional experiments, such as ATP determination assays for ATP synthases’ lactylation, and assessing sarcomere contractility after lactylation in structural proteins.

## 6. Conclusions

Our study presents the in-depth lactylome analysis of cardiac hypertrophy, revealing that Kla is not only involved in regulating energy metabolism but also in preserving the structural integrity of hypertrophic hearts. These findings suggest that Kla serves as a multifaceted regulator of cardiac remodeling, providing potential therapeutic targets for managing heart failure. Future research is required to fully elucidate the mechanisms by which Kla influences heart disease progression and to explore its potential for novel treatments.

## Figures and Tables

**Table 1:** Differentially lactylated proteins and sites in mouse cardiac hypertrophy models.

Gene Name	Protein Accession	Protein Description	Subcellular Location	Previously Reported Genes Associated with LVH (Yes/No)	HCM Pathogenic Genes (Yes/No)	Differentially Kla-Modified Sites	
						Up	Down
Acaa2	Q8BWT1	3-ketoacyl-CoA thiolase	Mitochondria	Yes (PMID: 20110695)	No	K214	-
Acad10	Q8K370	Acyl-CoA dehydrogenase family member	Mitochondria	Yes (PMID: 26669660)	No	K1056	-
Acadm	P45952	Medium-chain specific acyl-CoA dehydrogenase	Mitochondria	Yes (PMID: 33589646)	No	K212	K179
Acadvl	P50544	Very long-chain specific acyl-CoA dehydrogenase	Mitochondria	No	No	K277	-
Aco2	Q99KI0	Aconitate hydratase	Mitochondria	No	No	K31 K409	-
Acot1	O55137	Acyl-coenzyme A thioesterase 1	Cytoplasm	No	No	K42 K304	-
Actn2	Q9JI91	Alpha-actinin-2	Sarcomere	Yes	Yes (PMID: 34526680; PMID: 33983830)	K433	-
Ak1	Q9R0Y5	Adenylate kinase isoenzyme 1	Cytoplasm	Yes (PMID: 33498641)	No	K27	-
Alb	P07724	Albumin	Extracellular	Yes (PMID: 10728348)	No	K212 K223 K376 K588	-
Atp2a2	O55143	Sarcoplasmic/endoplasmic reticulum calcium ATPase 2	Sarcoplasmic reticulum	Yes (PMID: 33983830)	Yes (PMID: 33983830)	K502	-
Atp5f1a	Q03265	ATP synthase subunit alpha	Mitochondria	Yes (PMID: 20483212)	No	K176 K239 K427 K503 K531	-
Atp5f1b	P56480	ATP synthase subunit beta	Mitochondria	No	No	K522	-
Atp5f1c	Q91VR2	ATP synthase subunit gamma	Mitochondria	No	No	K83	K138
Atp5mf	P56135	ATP synthase subunit f	Mitochondria	No	No	-	K8
Atp5pb	Q9CQQ7	ATP synthase F(0) complex subunit B1	Mitochondria	No	No	K249	-
Atp5pf	P97450	ATP synthase-coupling factor 6	Mitochondria	No	No	K79	-
Auh	Q9JLZ3	Methylglutaconyl-CoA hydratase	Mitochondria	Yes (PMID: 7691603)	No	K75	-
Bola3	Q8CEI1	BolA-like protein 3	Mitochondria	No	No	-	K76
Cox4i1	P19783	Cytochrome c oxidase subunit 4 isoform 1	Mitochondria	Yes (PMID: 33589646)	No	K60	-
Cryab	P23927	Alpha-crystallin B chain	Cytoplasm	Yes (PMID: 33983830)	Yes (PMID: 33983830)	K174	-
Cs	Q9CZU6	Citrate synthase	Mitochondria	Yes (PMID: 3159381)	No	K103 K321 K370 K450	K366
Csrp3	P50462	Cysteine and glycine-rich protein 3	Sarcomere	Yes (PMID: 34526680; PMID: 33983830)	Yes (PMID: 34526680; PMID: 33983830)	K9 K109 K119 K113	-
Decr1	Q9CQ62	2,4-dienoyl-CoA reductase [(3E)-enoyl-CoA-producing]	Mitochondria	Yes (PMID: 34262469)	No	K106 K260	-
Des	P31001	Desmin	Sarcomere	Yes (PMID: 34526680)	Yes (PMID: 34526680)	K298	-
Dld	O08749	Dihydrolipoyl dehydrogenase	Mitochondria	Yes (PMID: 36572672)	No	K159	-
Dlst	Q9D2G2	Dihydrolipoyllysine-residue succinyltransferase component of 2-oxoglutarate dehydrogenase complex	Mitochondria	Yes (PMID: 28611091)	No	K155	-
Echs1	Q8BH95	Enoyl-CoA hydratase	Mitochondria	No	No	K101	-
Eci1	P42125	Enoyl-CoA delta isomerase 1	Mitochondria	No	No	K76	
Eno3	P21550	Beta-enolase	Cytoplasm	Yes (PMID: 18993053)	No	-	K80
Etfa	Q99LC5	Electron transfer flavoprotein subunit alpha	Mitochondria	No	No	K331	K62 K69
Fabp3	P11404	Fatty acid-binding protein	Cytoplasm	Yes (PMID: 28231848)	No	K15	-
Fh	P97807	Fumarate hydratase	Mitochondria	No	No	K169 K170	-
Gatd3a	Q9D172	Glutamine amidotransferase-like class 1 domain-containing protein 3A	Mitochondria	No	No	K155 K231	-
Got2	P05202	Aspartate aminotransferase	Mitochondria	Yes (PMID: 31084377)	No	K396 K404	-
Hadh	Q61425	Hydroxyacyl-coenzyme A dehydrogenase	Mitochondria	Yes (PMID: 23709660)	No	K81	-
Hadha	Q8BMS1	Trifunctional enzyme subunit alpha	Mitochondria	Yes (PMID: 19265432)	No	K262 K625	-
Hadhb	Q99JY0	Trifunctional enzyme subunit beta	Mitochondria	No	No	K189 K272 K292	-
Hba	P01942	Hemoglobin subunit alpha	Extracellular	No	No	K8 K57	-
Hbb-b1	P02088	Hemoglobin subunit beta-1	Extracellular	No	No	K67	-
Hspa9	P38647	Stress-70 protein	Mitochondria	No	No	K345	-
Hspd1	P63038	60 kDa heat shock protein	Mitochondria	No	No	-	K292
Idh2	P54071	Isocitrate dehydrogenase [NADP]	Mitochondria	Yes (PMID: 25557279)	No	K133 K280	K384
Idh3a	Q9D6R2	Isocitrate dehydrogenase [NAD] subunit alpha	Mitochondria	Yes (PMID: 35920168)	No	K223	-
Immt	Q8CAQ8	MICOS complex subunit Mic60	Mitochondria	No	No	K102	K450
Ldb3	Q9JKS4	LIM domain-binding protein 3	Sarcomere	Yes (PMID: 34158421)	No	K218	K507
Letm1	Q9Z2I0	Mitochondrial proton/calcium exchanger protein	Mitochondria	No	No	K458	-
Mb	P04247	Myoglobin	Sarcoplasmic plasma	Yes (PMID: 6085307)	No	K51 K57 K97	-
Mccc1	Q99MR8	Methylcrotonoyl-CoA carboxylase subunit alpha	Mitochondria	No	No	K233	-
Mdh2	P08249	Malate dehydrogenase	Mitochondria	Yes (PMID: 3159381)	No	K157 K335	-
Mrpl12	Q9DB15	39S ribosomal protein L12	Mitochondria	No	No	K145	-
Mybpc3	O70468	Myosin-binding protein C	Sarcomere	Yes (PMID: 34526680; PMID: 33983830; PMID: 33215938)	Yes (PMID: 34526680; PMID: 33983830; PMID: 33215938)	K193	-
Myh6	Q02566	Myosin-6	Sarcomere	Yes (PMID: 33983830;)	Yes (PMID: 33983830;)	K1175 K922 K1326 K58 K1786 K1581 K1840 K72 K1307 K1773 K1897 K878 K1793 K1643 K1533 K1056 K867 K1249 K914 K566 K1921 K1446 K1264	K912
Myh7	Q91Z83	Myosin-7	Sarcomere	Yes (PMID: 34526680; PMID: 33983830; PMID: 33215938)	Yes (PMID: 34526680; PMID: 33983830; PMID: 33215938)	-	K942
Myh7b	A2AQP0	Myosin-7B	Sarcomere	Yes (PMID: 32207065)	Yes (PMID: 32207065)	K958 K959	-
Myl2	P51667	Myosin regulatory light chain 2	Sarcomere	Yes (PMID: 33983830; PMID: 33215938)	Yes (PMID: 33983830; PMID: 33215938)	K62	K91
Myl3	P09542	Myosin light chain 3	Sarcomere	Yes (PMID: 33983830; PMID: 33215938)	Yes (PMID: 33983830; PMID: 33215938)	K19 K109	-
Ndufa4	Q62425	Cytochrome c oxidase subunit NDUFA4	Mitochondria	No	No	K10	-
Ndufb3	Q9CQZ6	NADH dehydrogenase [ubiquinone] 1 beta subcomplex subunit 3	Mitochondria	No	No	-	K40
Ndufs3	Q9DCT2	NADH dehydrogenase [ubiquinone] iron-sulfur protein 3	Mitochondria	Yes (PMID: 24388463)	No	K258	-
Ndufs8	Q8K3J1	NADH dehydrogenase [ubiquinone] iron-sulfur protein 8	Mitochondria	No	No	K42 K51	-
Ndufv2	Q9D6J6	NADH dehydrogenase [ubiquinone] flavoprotein 2	Mitochondria	No	No	-	K158
Nebl	Q9DC07	LIM zinc-binding domain-containing Nebulette	Sarcomere	Yes (PMID: 33861145)	No	K148	-
Nipsnap2	O55126	Protein NipSnap homolog 2	Mitochondria	No	No	K53	-
Ogdh	Q60597	2-oxoglutarate dehydrogenase	Mitochondria	Yes (PMID: 31146816)	No	K534	-
Oxct1	Q9D0K2	Succinyl-CoA:3-ketoacid coenzyme A transferase 1	Mitochondria	No	No	K446	-
Pdha1	P35486	Pyruvate dehydrogenase E1 component subunit alpha	Mitochondria	No	No	K83 K313	-
Pfkm	P47857	ATP-dependent 6-phosphofructokinase	Cytoplasm	Yes (PMID: 27312951)	No	K678	-
Sdha	Q8K2B3	Succinate dehydrogenase [ubiquinone] flavoprotein subunit	Mitochondria	No	No	K485	-
Slc25a11	Q9CR62	Mitochondrial 2-oxoglutarate/malate carrier protein	Mitochondria	No	No	K256	-
Slc25a4	P48962	ADP/ATP translocase 1	Mitochondria	Yes (PMID: 23401503)	No	K137	K63
Slc25a5	P51881	ADP/ATP translocase 2	Mitochondria	No	No	-	K166
Sucla2	Q9Z2I9	Succinate-CoA ligase [ADP-forming] subunit beta	Mitochondria	No	No	K139 K143	-
Suclg1	Q9WUM5	Succinate-CoA ligase [ADP/GDP-forming] subunit alpha	Mitochondria	No	No	K48 K90 K94 K308	-
Suclg2	Q9Z2I8	Succinate-CoA ligase [GDP-forming] subunit bet	Mitochondria	No	No	K74	-
Tnni3	P48787	Troponin I	Sarcomere	Yes (PMID: 33983830; PMID: 33215938)	Yes (PMID: 33983830; PMID: 33215938)	K107	-
Tnnt2	P50752	Troponin T	Sarcomere	Yes (PMID: 33983830; PMID: 33215938)	Yes (PMID: 33983830; PMID: 33215938)	K137 K230	-
Tpm1	P58771	Tropomyosin alpha-1 chain	Sarcomere	Yes (PMID: 33983830; PMID: 33215938)	Yes (PMID: 33983830; PMID: 33215938)	K48 K65 K77 K137 K152 K161 K168	-
Trim72	Q1XH17	Tripartite motif-containing protein 72	Cytoplasm	Yes (PMID: 30820965)	No	K398	-
Ttn	A2ASS6	Titin	Sarcomere	Yes (PMID: 34526680; PMID: 33983830)	Yes (PMID: 34526680; PMID: 33983830)	K477 K698 K2029 K2502 K13401 K22412	-
Uqcrfs1	Q9CR68	Cytochrome b-c1 complex subunit Rieske	Mitochondria	No	No	K101	-

Abbreviations: Kla = lysine lactylation; LVH = left ventricular hypertrophy, HCM = hypertrophic cardiomyopathy.

## Data Availability

The mass spectrometry proteomics data have been deposited to the ProteomeXchange Consortium (https://proteomecentral.proteomexchange.org/, accessed on 16 June 2026) via the PRIDE partner repository with the dataset identifier PXD061494.

## Supplementary Materials

The following supporting information can be downloaded at: https://www.mdpi.com/article/10.3390/jcdd13070297/s1, Figure S1: Myocardial hypertrophy in vitro cardiomyocytes after treatment with 50μM phenylephrine; Figure S2: Sample reproducibility was evaluated using Pearson’s Correlation Coefficient (PCC), Principal Component Analysis (PCA), and Relative Standard Deviation (RSD); Figure S3: The data quality control analysis; Figure S4: Enrichment Analysis of Kla-Modified Proteins in Cellular Processes, Components, and Functions (overall); Figure S5: The COG/KOG functional analysis of differentially Kla-modified proteins (overall); Figure S6: Enrichment Analysis of Kla-Modified Proteins in Cellular Processes, Components, and Functions (upregulated Kla modified proteins); Figure S7: Enrichment Analysis of Kla-Modified Proteins in Cellular Processes, Components, and Functions (downregulated Kla modified proteins); Figure S8: The COG/KOG functional analysis of differentially Kla-modified proteins (upregulated Kla modified proteins); Figure S9: The COG/KOG functional analysis of differentially Kla-modified proteins (downregulated Kla modified proteins); Figure S10: GO functional enrichment analysis of Kla proteins in cardiac hypertrophy based on biological processes (A), molecular functions (D), and cellular components (C); Figure S11: KEGG functional enrichment analysis of Kla proteins; Figure S12: GO and KEGG Functional Enrichment of Kla-modified Proteins in Cardiac Hypertrophy. Figure S13: Protein-protein interaction network highlighting key proteins in metabolic and cardiac processes.

## Abbreviations

Kla	Lysine lactylation
PTM	Post-translational modification
PPI	Protein–protein interaction
TCA	Tricarboxylic acid
AB	Aortic banding
WT	Wild-type
BCA	Bicinchoninic acid
SDS-PAGE	Sodium dodecyl sulfate-polyacrylamide gel electrophoresis
PVDF	Polyvinylidene difluoride
TBS-T	Tris-buffered saline with Tween-20
LC-MS/MS	Liquid chromatography with tandem mass spectrometry
UHPLC	Ultra-high-performance liquid chromatography
FDR	False discovery rate
GO	Gene Ontology
KEGG	Kyoto Encyclopedia of Genes and Genomes
COG/KOG	Clusters of Orthologous Groups/Eukaryotic Orthologous Groups
PCA	Principal component analysis
RSD	Relative standard deviation
FC	Fold change
AARS1	Alanyl-tRNA synthetase 1
HBO1	Histone Acetyltransferase 1
KAT8	Lysine Acetyltransferase 8
NBS1	Nijmegen Breakage Syndrome 1
TEAB	Triethylammonium bicarbonate
