# Supplementary material for: Global Profiling of Protein Lysine Lactylation in Mouse Cardiac Hypertrophy: A Lactylome Analysis
Source: J Cardiovasc Dev Dis. 2026 Jun 29;13(7):297. doi: 10.3390/jcdd13070297 (PMC13409870; doi:10.3390/jcdd13070297)
Supplement: Supplementary file 1 [file jcdd-13-00297-s001.zip › jcdd-4279601-supplementary.pdf]

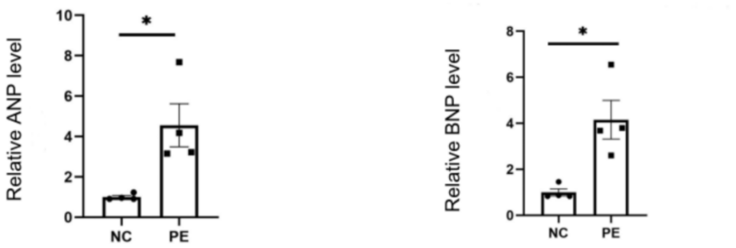

Supplementary Figure S1. Myocardial hypertrophy in vitro cardiomyocytes after treatment with 50μM phenylephrine

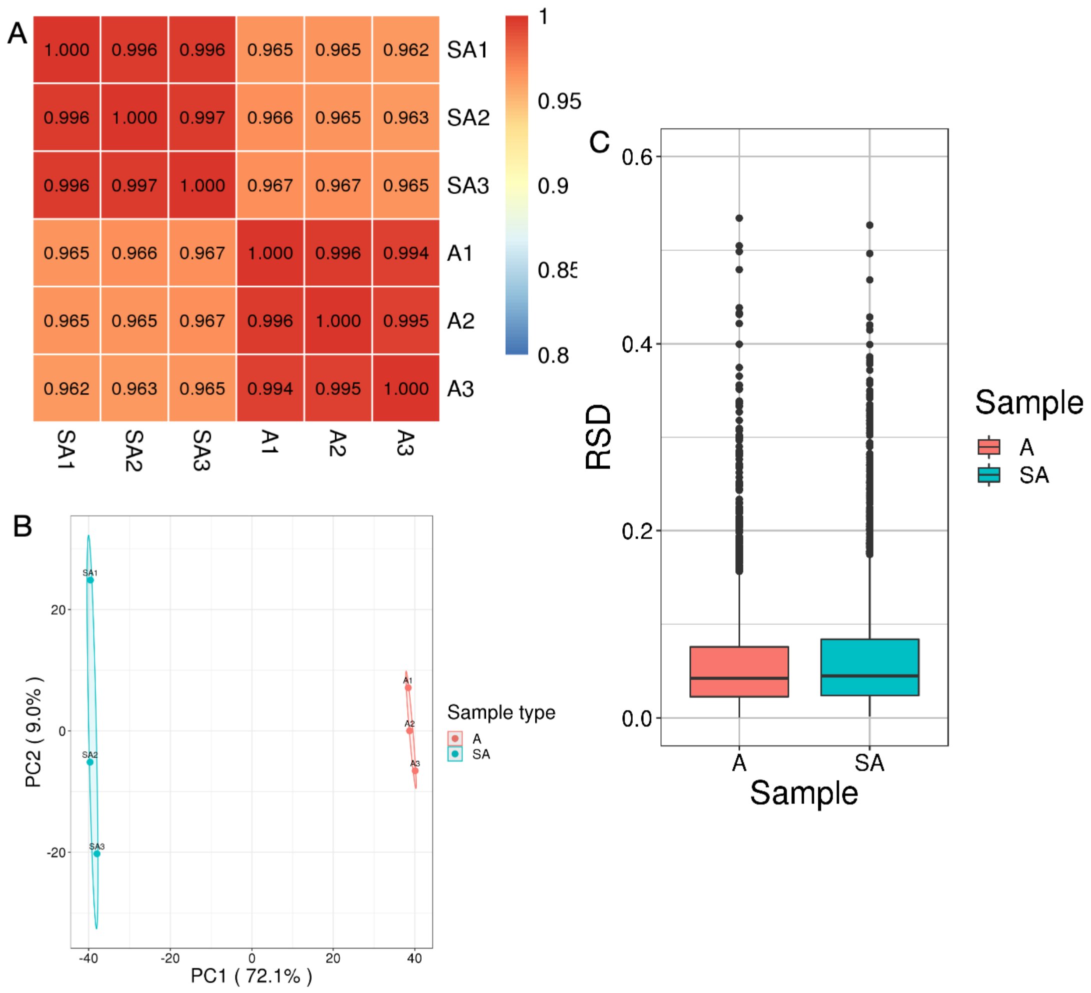

Supplementary Figure S2. Sample reproducibility was evaluated using Pearson's Correlation Coefficient (PCC), Principal Component Analysis (PCA), and Relative Standard Deviation (RSD). A) *The PCC heatmap shows high correlation between replicates, indicating good consistency.* **B)** *The PCA plot reveals clear clustering of samples within groups, demonstrating low variability.* **C)** *The RSD boxplot further confirms the reproducibility, with low RSD values indicating high precision in the quantification across sample groups. A indicates 4-week AB mice, SA indicates sham-operated WT mice.*

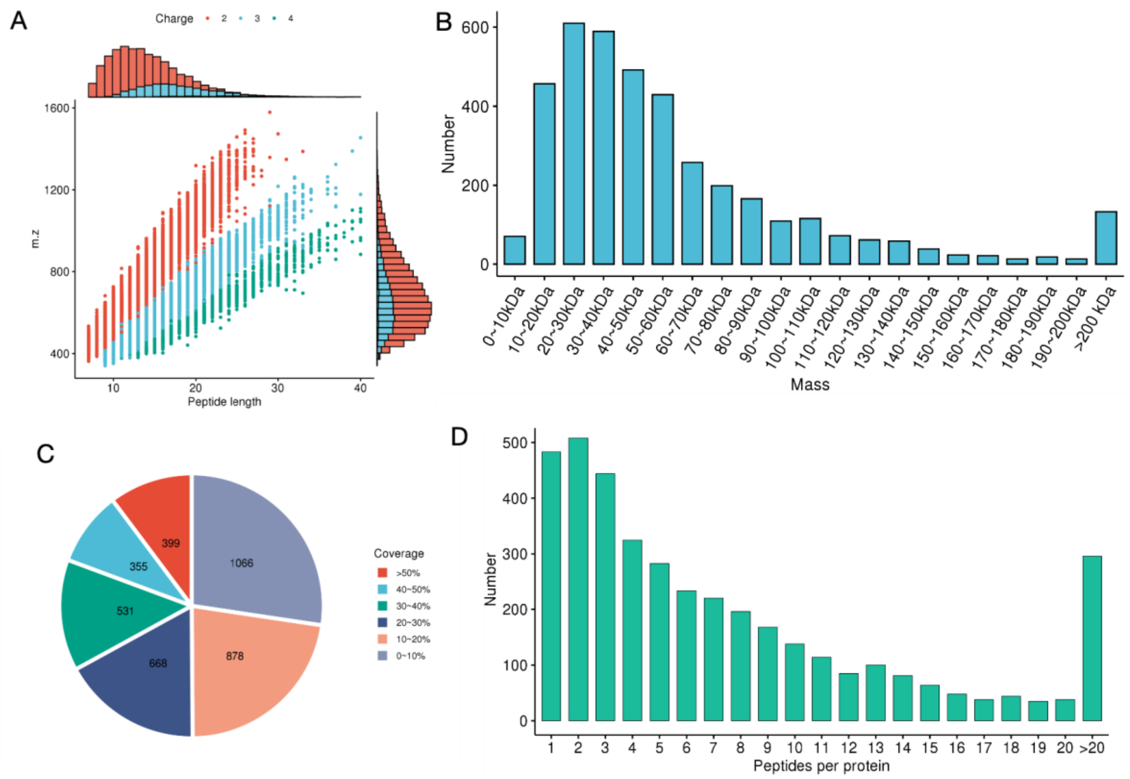

Supplementary Figure S3. The data quality control analysis. *A) Peptide length distribution: most peptides are between 7-20 amino acids, which is consistent with typical tryptic digestion. B) Peptides per protein distribution: this graph illustrates the number of peptides identified per protein, with most proteins associated with two or more peptides, enhancing quantification reliability. C) Protein coverage distribution: the protein coverage distribution indicates that most proteins have a coverage below 30%, which is common in shotgun proteomics analyses. D) Protein molecular weight distribution: the molecular weight distribution of identified proteins shows a wide range, reflecting the diversity of proteins detected in the analysis.*

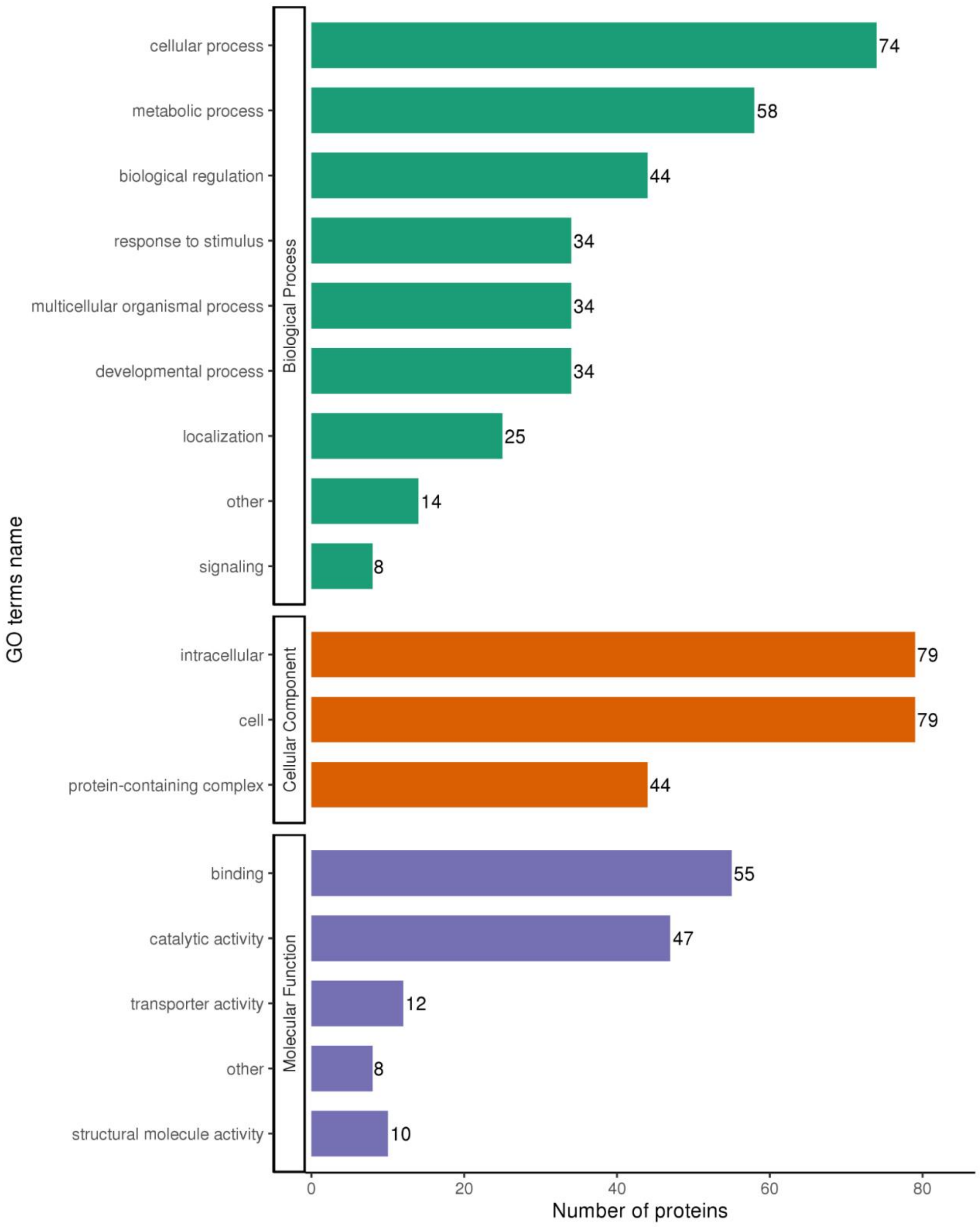

Supplementary Figure S4. Enrichment Analysis of Kla-Modified Proteins in Cellular Processes, Components, and Functions (overall)

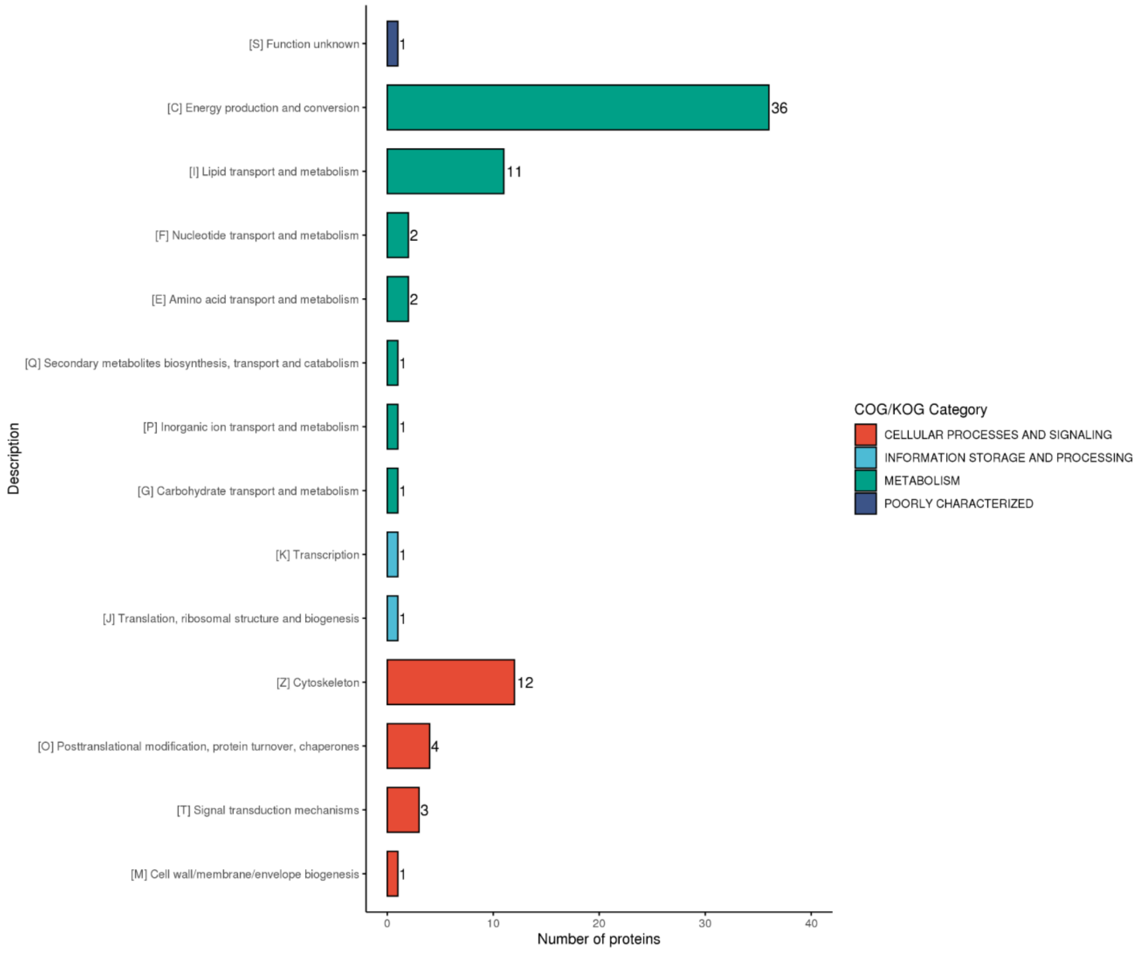

Supplementary Figure S5. The COG/KOG functional analysis of differentially Kla-modified proteins (overall)

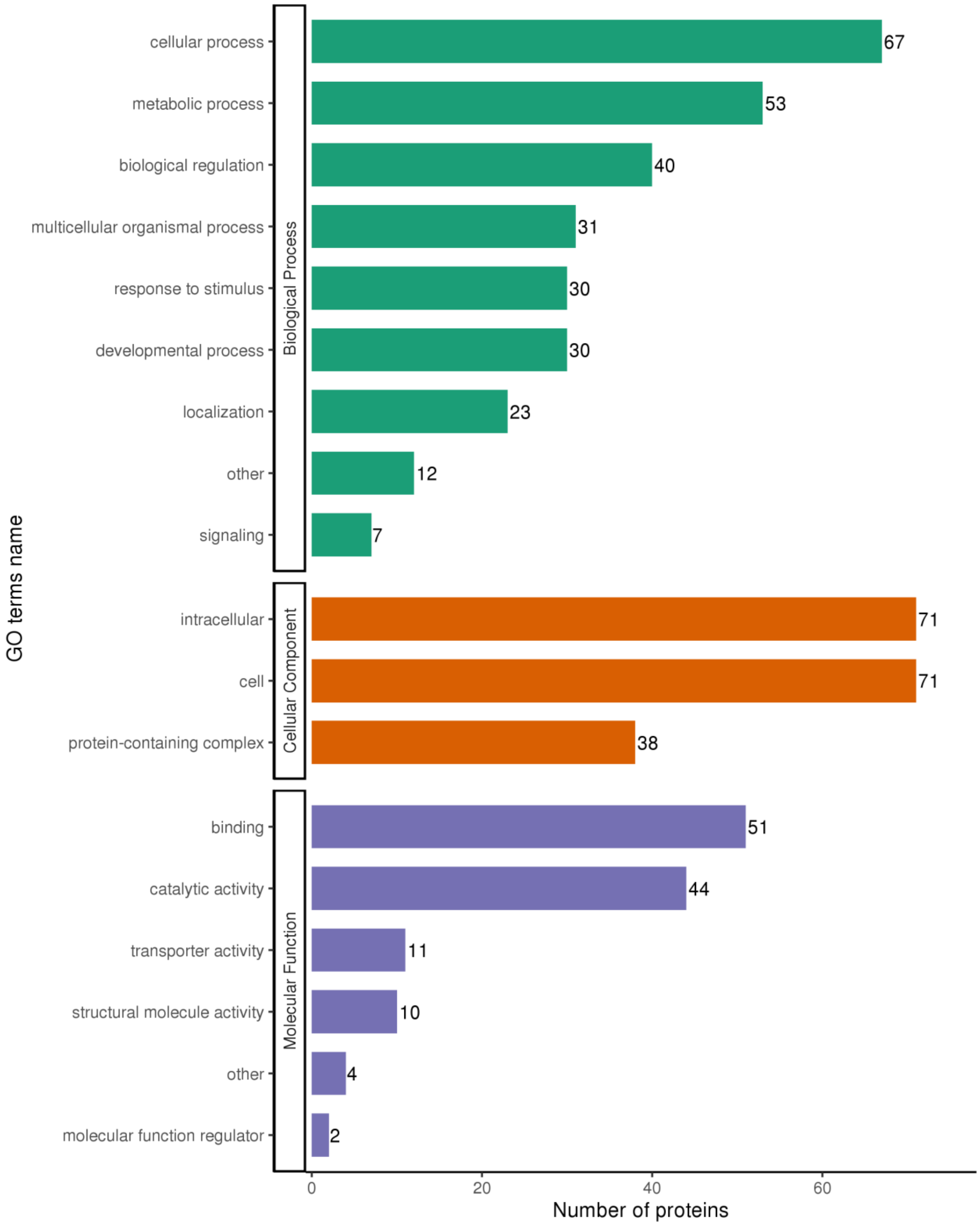

Supplementary Figure S6. Enrichment Analysis of Kla-Modified Proteins in Cellular Processes, Components, and Functions (upregulated Kla modified proteins)

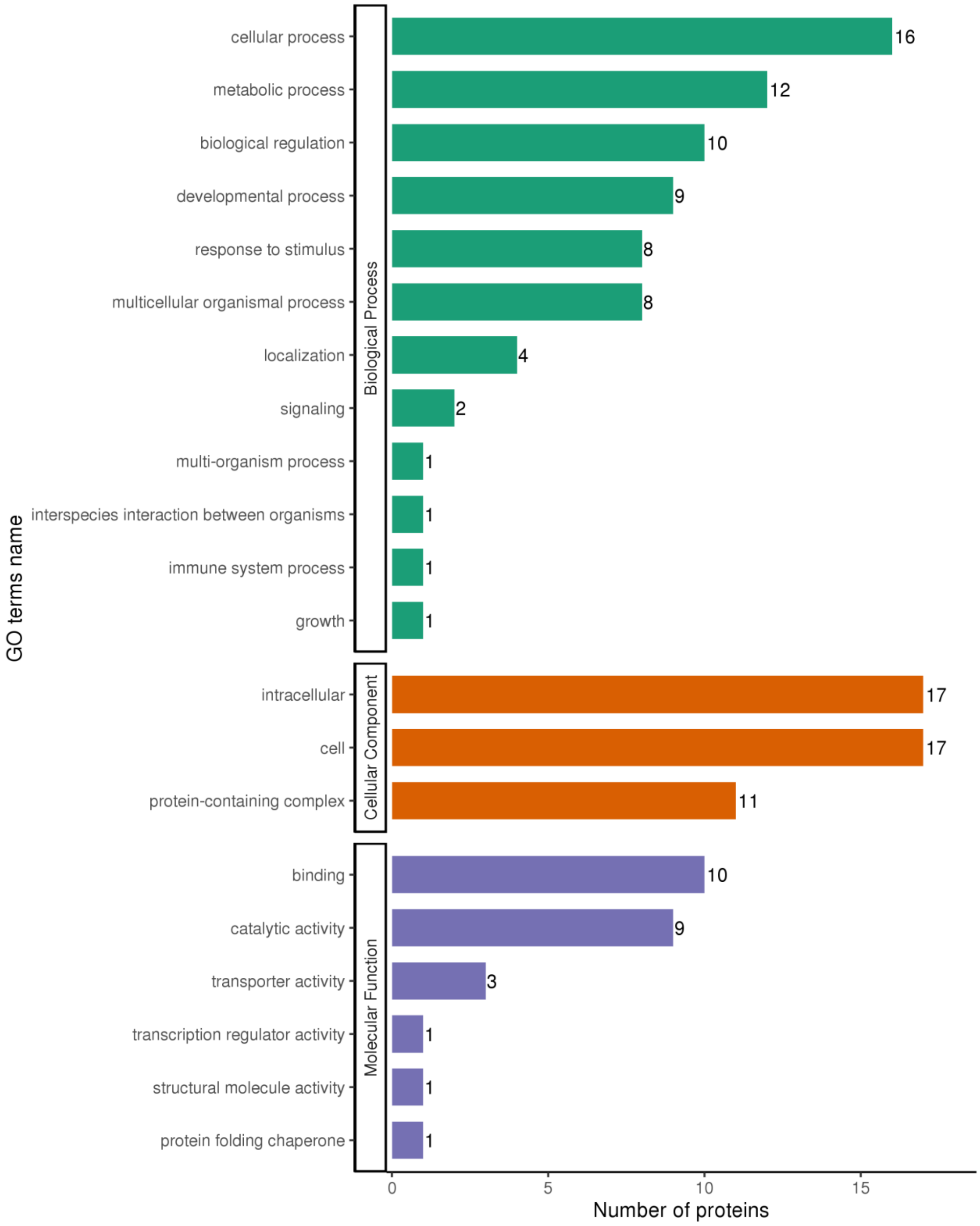

Supplementary Figure S7. Enrichment Analysis of Kla-Modified Proteins in Cellular Processes, Components, and Functions (downregulated Kla modified proteins)

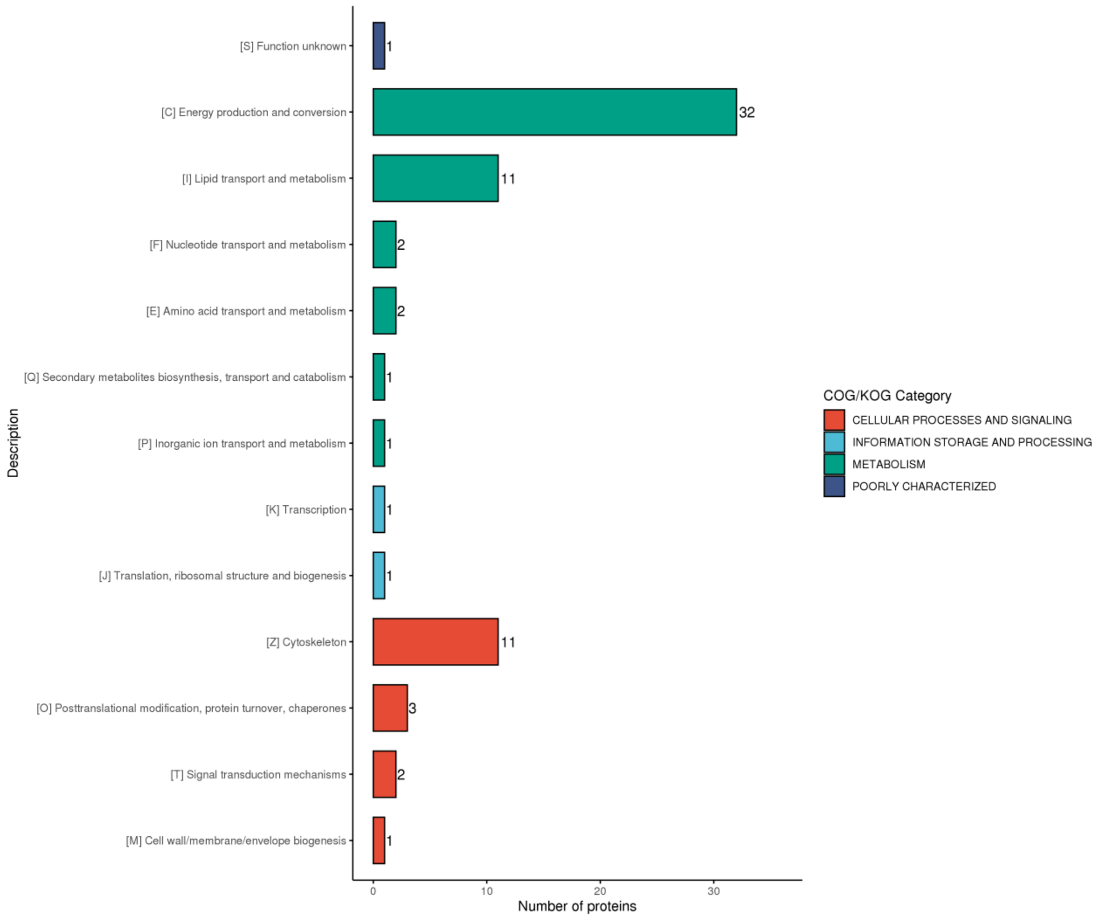

Supplementary Figure S8. The COG/KOG functional analysis of differentially Kla-modified proteins (upregulated Kla modified proteins)

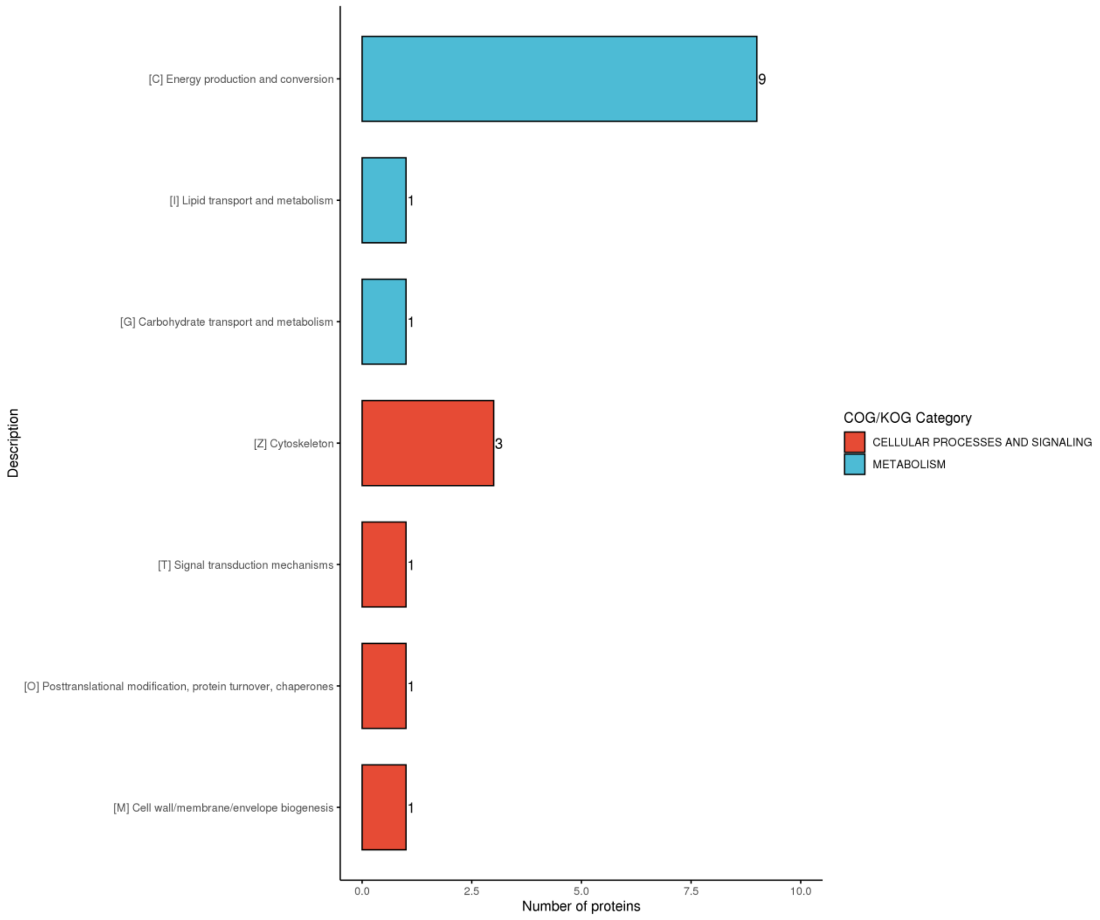

Supplementary Figure S9. The COG/KOG functional analysis of differentially Kla-modified proteins (downregulated Kla modified proteins)

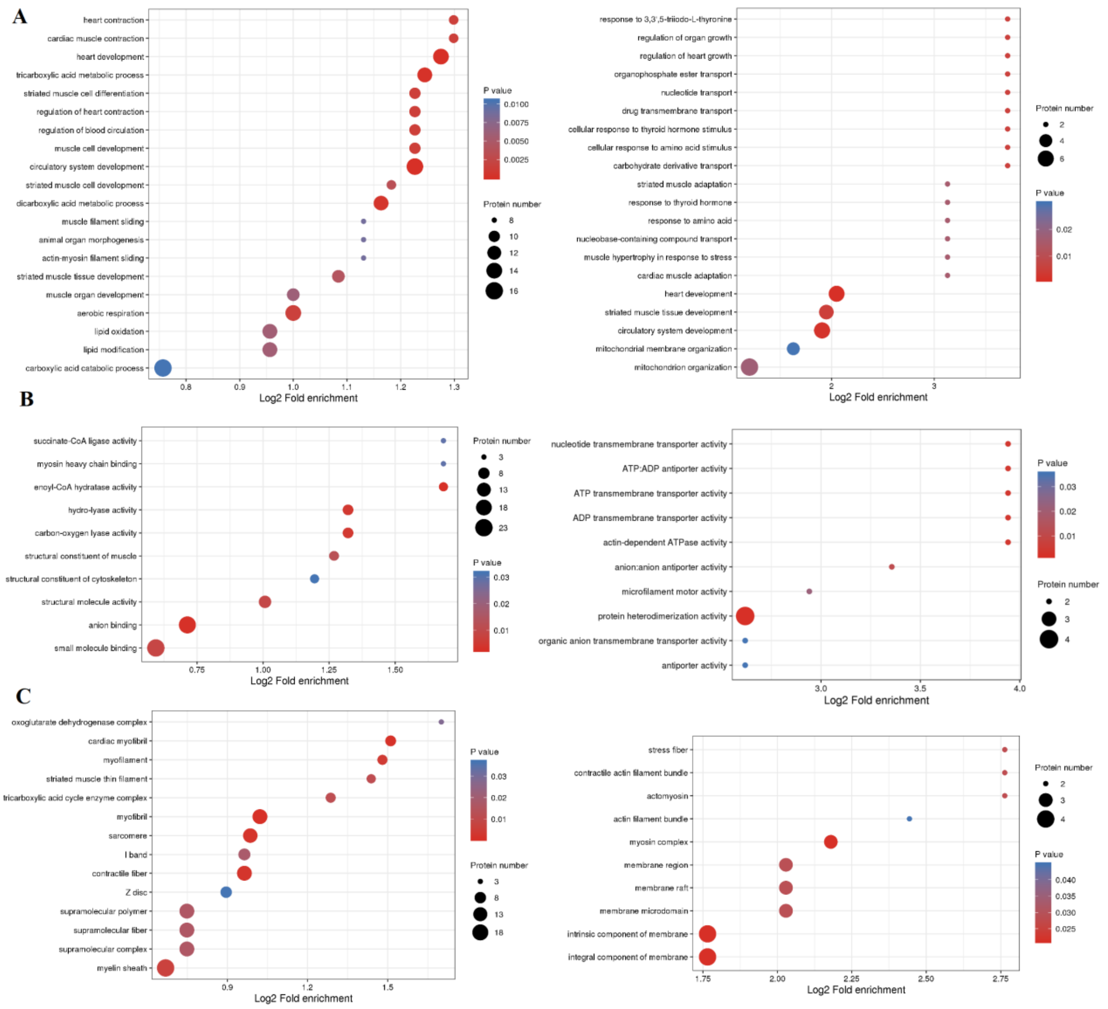

Supplementary Figure S10. GO functional enrichment analysis of Kla proteins in cardiac hypertrophy based on biological processes (A), molecular functions (D), and cellular components (C). Left panel: upregulated Kla modified proteins; Right panel: downregulated Kla modified proteins

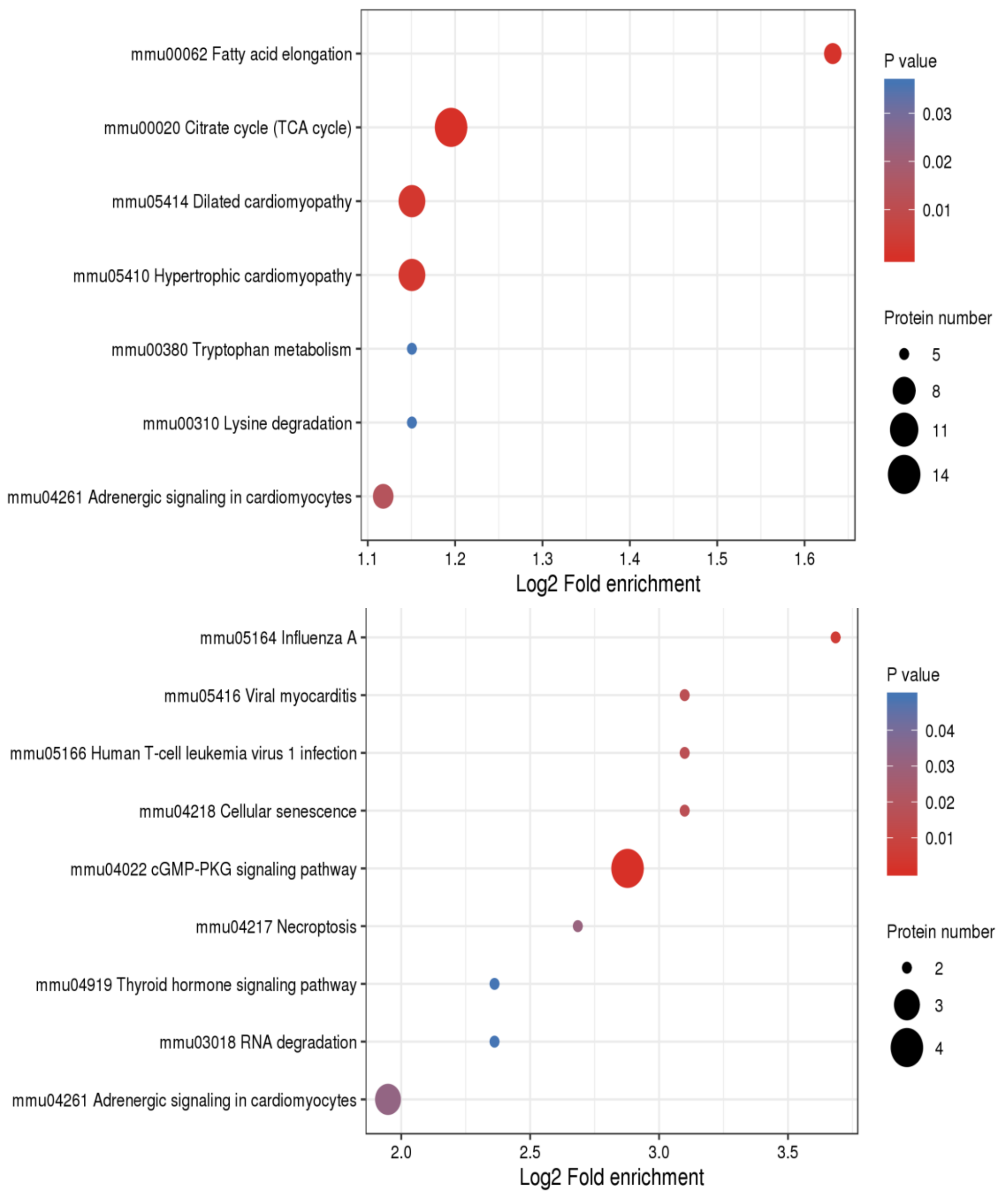

Supplementary Figure S11. KEGG functional enrichment analysis of Kla proteins. Up panel: upregulated Kla modified proteins; Down panel: downregulated Kla modified proteins

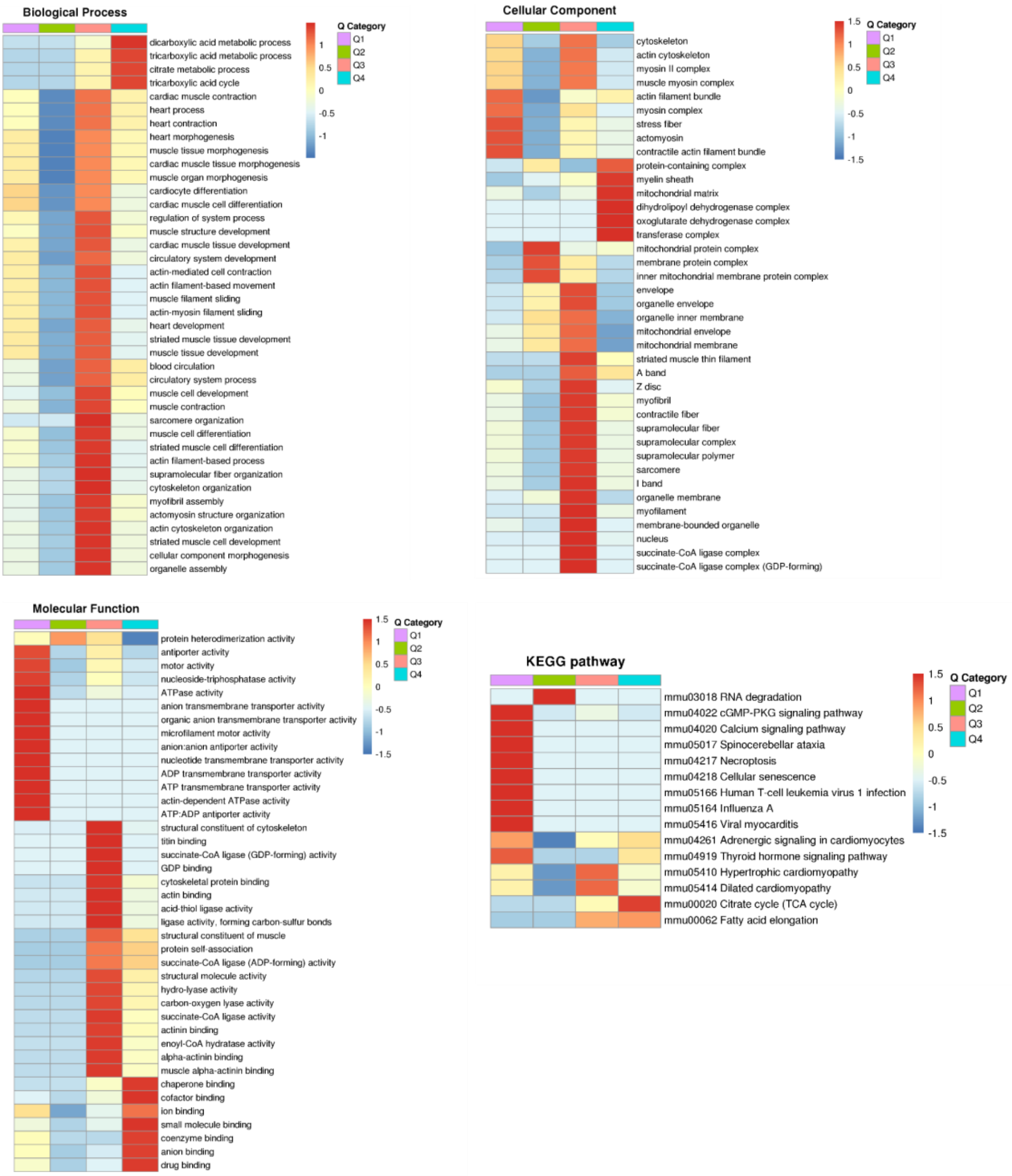

Supplementary Figure S12. GO and KEGG Functional Enrichment of Kla-modified Proteins in Cardiac Hypertrophy. Four clusters based on their expression levels: Q1: <0.5, Q2: 0.5–1/1.5, Q3: 1.5–2.0, Q4: >2.0.

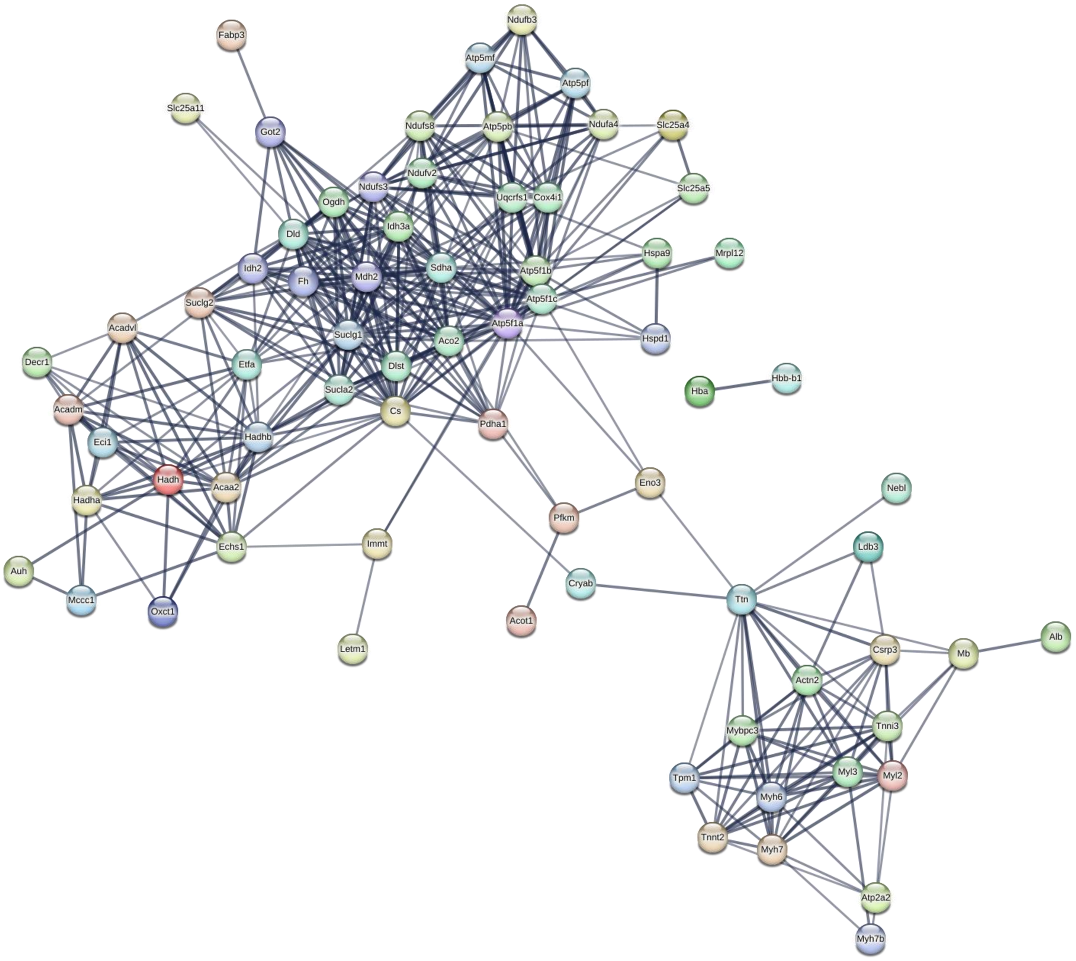

Supplementary Figure S13. Protein-protein interaction network highlighting key proteins in metabolic and cardiac processes.
